# Trends and geographic patterns of overweight and obesity among Tanzanian adults: Evidence from the 2010–2022 Demographic and Health Surveys

**DOI:** 10.1371/journal.pone.0332275

**Published:** 2025-09-22

**Authors:** Angelina Mageni Lutambi, Gerald Phares Mwing’a, Basiliana Emidi

**Affiliations:** Dodoma Medical Research Centre, National Institute for Medical Research, Dodoma, Tanzania; Africa Academy for Public Health, TANZANIA, UNITED REPUBLIC OF

## Abstract

Overweight and obesity are risk factors for several non-communicable diseases. In Tanzania, despite the increasing public health concern, detailed spatial information on the distribution of overweight and obesity is limited. This study aimed to determine the prevalence, show spatial and temporal variations and identify factors that impact overweight and obesity in Tanzania. We used cross-sectional survey secondary data from the Tanzanian Demographic and Health Surveys (DHS) which collected anthropometric measurements in women aged 15−49 years in 2010, and 2015−16, and both women and men in the 2022 survey. Spatial interpolation was performed to estimate prevalence at unsampled locations, while generalized additive models were used to identify factors and assess their effect on the spatial distribution of overweight and obesity risk. The study included 33,787 participants (9,029 in 2010, 11,940 in 2015−16, and 12,818 in 2022). The overall mean age was 29 (SD = 10) years. The prevalence of overweight and obesity among women increased by 45.45%, rising from 22% in 2010 to 32% in 2022, with higher rates observed in urban areas and among wealthier and more educated women. In 2022, women were disproportionately affected, with 32% being overweight or obese compared to 15% of men, and 45% were urban women and 23% urban men. Age and wealth index were consistent significant factors across all surveys while place of residence was a significant factor in 2010 and 2015. Geographic disparities were evident, with the eastern, southern highlands, and northern regions showing higher prevalence compared to the lake zone. Overweight and obesity are increasing in Tanzania, driven by wealth and age. Urban residence was a significant factor in early years and its influence declined in 2022. The observed regional disparities highlight the urgent need for targeted and multi-sectoral interventions.

## Background

Overweight and obesity pose a significant global health challenge, as they are major metabolic risk factors for numerous non-communicable diseases (NCDs) [1–3]. The burden of non-communicable diseases indicate that diseases such as diabetes, cardiovascular diseases, and cancers, are expected to increase significantly due to the growing burden of overweight and obesity [4]. By 2050, over 1.31 billion people are projected to develop diabetes [5], and cardiovascular and obesity-related cancers are also anticipated to increase [6,7]. Additionally, overweight and obesity increases the risk of death, contributing to approximately 4 million deaths globally [4]. Studies also show that overweight and obesity have increased significantly across all regions since 1990, though with notable differences between countries [4,8]. In 2021, more than 2.11 billion adults and 173.7 million children and adolescents were overweight or obese. Projections suggest that by 2050, over half of the global adult population (approximately 3.8 billion people) will be overweight or obese, and the number of obese children and adolescents is expected to rise to 361 million [9,10]. According to the Global Burden of Diseases (GBD) Study 2021, overweight and obesity ranked among the top ten leading risk factors, with their burden in terms of percentage change of age-standardised disability-adjusted life years rates estimated to have increased by 15.7% between 2000 and 2021 [11]. Due to this increasing burden and the corresponding health effects, urgent action is required to implement effective prevention strategies to address the escalating overweight and obesity related health concerns.

Like in many other countries, overweight and obesity are significant modifiable risk factors for several non-communicable diseases in Tanzania [12–14], and studies have shown that their prevalence is mounting [15–18]. However, evidence on their spatial distribution at finer local scales within Tanzania remains limited. Existing studies within the country have largely focused on specific locations without conducting comparative spatial analyses across the country [15–17] or have analyzed data within broader multiple country contexts [19]. These approaches often overlook the diversity of local population structures, socio-demographic characteristics, and varying levels of socio-economic status that influence life style, leading to variability in risks, consequently, health outcomes.

Socio-demographic variables are essential in studying overweight and obesity due to the fact that they capture the complex social and economic contexts that influence these conditions. While biological and genetic factors contribute to overweight and obesity, the increasing global prevalence and its variation across regions highlight the significancy of demographic and socio-economic determinants. These factors shape health behaviours related to diet and physical activity levels [20–27]. Several studies have established socio-economic and demographic factors as key determinants of overweight and obesity. Evidence from low-and middle-income countries have demonstrated that individuals who are older, more educated, wealthier, or living in urban areas are at a higher risk of being overweight or obese [28–32]. Sex also plays a significant role, with women generally showing higher prevalence rates of excess body weight compared to men [9,10].

Understanding spatial disparities in overweight and obesity and how these socio-economic and demographic characteristics influence risk differences at finer scales is essential for designing targeted and effective public health interventions. Our study addresses this gap by using spatial modelling approaches designed for subnational analyses [33,34]. Specifically, we used adaptive kernel density estimation methods to estimate subnational prevalence and generalized additive models (GAMs) to generate risk maps of overweight and obesity. By applying these techniques, we generated continuous surface estimates of prevalence of overweight and obesity and assessed the influence of associated factors across the country. This approach allowed capturing local variations in the distribution of this key NCD risk factor. Therefore, the primary objective of this study was to estimate prevalence of overweight and obesity at subnational level, show spatial and temporal variations, identify high risk areas, and assess the contribution of factors that impact overweight and obesity in Tanzania.

## Materials and methods

### Study setting

This study was conducted in Tanzania, a country located south of the equator along the eastern coast of the Indian Ocean. The majority of the country lies above 200 metres in elevation, with significant geographical diversity. Administratively, Tanzania is divided into regions and further grouped into nine geographical zones based on shared characteristics such as weather patterns and climate. The country experiences a tropical climate with distinct dry and rainy seasons. The dry season extends from May to October, while the rainy season typically occurs from November to May. Rainfall distribution varies across regions, influencing agricultural productivity and contributing to differences in food availability and nutritional outcomes. These geographical and climatic variations are important factors in understanding the distribution and determinants of overweight and obesity across the country. Additionally, the country’s urbanization rate is estimated at about 5% [35], reflecting ongoing transitions in diet and health behaviours associated with urban lifestyle.

### Data and study design

We used secondary data from the Demographic and Health Surveys (DHS) managed by the DHS program [36]. The DHS surveys are cross-sectional studies that collect several health indicators from the general population and have been implemented in Tanzania. The Tanzania DHS 2010 and 2015−16 surveys collected anthropometric data on women aged 15−49 [37,38], while the 2022 survey included both women and men in the same age group [39]. This enabled a sex-stratified analysis in 2022 to explore sex-specific differences in overweight and obesity, while allowing for temporal comparisons using the female subsample across all three survey years. The surveys sampled 475, 608, and 629 clusters, respectively, and were conducted between December 2009 and May 2010 for the DHS 2010, from August 2015 to February 2016 for the DHS 2015−16, and between February to July 2022 for the DHS 2022. These surveys followed a two-stage stratified sampling design. In the first stage, clusters were selected consisting of Census enumeration areas. These enumeration areas were selected with a probability proportional to their size within each sampling stratum. In the second stage, households were selected from each cluster in which all household members meeting the eligibility criteria were included. To allow for spatial analysis, the DHS georeferenced each sampled cluster at its centroid location at an accuracy of less than 15 meters. Georeferencing of the clusters was then randomly displaced for confidentiality, with urban clusters displacements of up to 2 kilometers and up to 10 kilometers for rural clusters.

### Data extraction and quality checks

**Fig 1:**
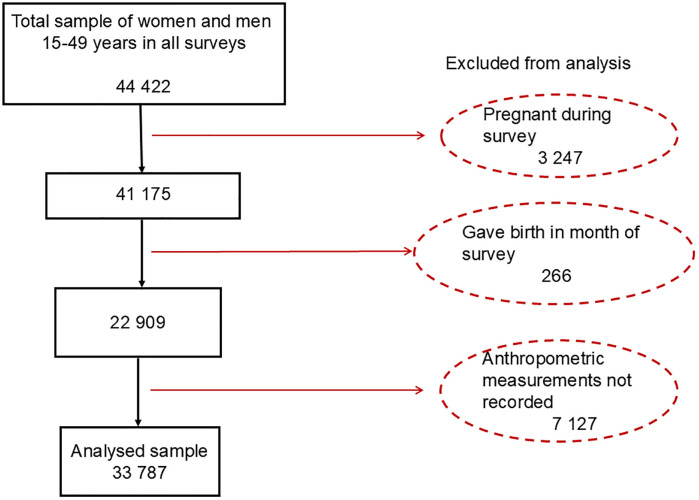
Flow diagram showing study sample screening.

We extracted datasets from the Tanzania DHSs for the years 2010, 2015−16, and 2022, which included individual recode files, household level data, and geospatial data. The datasets were pre-processed, cleaned, and recoded to ensure consistency. Initial quality checks were conducted through tabulations to identify missing data and inconsistencies. A total of 33,787 participants aged 15–49 years with recorded anthropometric measurements were included in the analysis (Fig 1), comprising 9,029 participants from the 2010 survey, 11,940 from 2015−16, and 12,818 from the 2022 survey.

### Outcome and other study variables

The outcome variable in this study was overweight and obesity, defined as Body mass index (BMI) ≥ 25 kg/m^2^. Overweight and obesity in the DHS surveys were derived from weight and height measurements for both women and men aged between 15–49 years using a SECA model 878U digital scale and a ShorrBoard® measuring board, respectively. The Body mass index was computed in accordance with World Health Organization (WHO) definitions, where those aged between 15 and 19 years, BMI-for-age, the ratio of weight relative to height for different age groups was used to measure nutritional status [40]. While for those aged between 20 and 49 years, BMI was computed as the ratio of weight relative to height squared. Like in many other studies [1,2], here, individuals with BMI ≥ 25 kg/m^2^ were categorized as overweight and obese. We combined overweight and obesity into a single outcome to ensure enough statistical power in the analysis. In addition, they represent a continuum of excess body weight, with individuals potentially transitioning between the two over time. Pregnant women and women who gave birth during the month of interview were excluded in the analysis.

Since socio-economic and demographic factors often intersect to influence health behaviours particularly those related to diet and physical activity levels [20–24], The Tanzania DHS have consistently been collecting these factors across all three surveys, allowing for temporal comparisons. These variables included age (categorized as 15–19, 20–29, 30–39 and 40–49), type of residence (urban, Rural), marital status (never, in union, separated), wealth index derived from household asset ownership (poorest, poorer, middle, richer, and richest), education level (none, primary, secondary, higher), and occupation type (none, not manual, manual, other). For women, studies have reported contraceptive use as an important factor that may influence body weight [41]. Since data on contraceptive use were collected only for women, this variable was included in the analysis for women only.

### Descriptive analysis

We conducted descriptive data analysis to obtain summary statistics of the study participants. We examined the distribution of overweight and obesity of the participants across all socio-demographic factors using cross tabulation. The results of the cross-tabulations were presented as percentages and their corresponding p-values derived from Pearson Chi-square for comparing categories within variables. Analysis was stratified by sex (where applicable) to examine any differences between women and men. The prevalence of overweight and obesity at each cluster was calculated as follows:


$$P(i,j)=\;\frac{Y_{T}(i,j)}{N_{T}(i,j)}\times \text{1}\text{0}\text{0}\%$$

where P(i,j) is the prevalence of overweight and obesity at cluster *i,j,* $Y_{T}(i,j)$ is the weighted number of sampled individuals who have overweight or obesity, and $N_{T}(i,j)$ is the weighted total number of people sampled at cluster *i,j*. To adjust for the impact of the complex sampling design, sample weights were applied in accordance with the DHS Guide to Statistics [42].

### Subnational prevalence estimation

To obtain prevalence estimates of overweight and obesity to unsampled locations across the country, we used *PrevR* package [33,43] to interpolate the measured prevalence. This interpolation method uses features within a neighbourhood to estimate values at locations with unmeasured prevalence. The method was designed to perform interpolation using data from DHS surveys that uses a stratified two-stage sampling design. It takes into account sampling weights to adjust for the impact of the complex sampling design as stipulated by the DHS statistics guideline [42] and the method has been used elsewhere in health research [19,43,44]. To examine the temporal changes in the spatial distribution of overweight and obesity, we identified areas with high prevalence for each survey. We combined the continuous surface prevalence estimates and calculated the 80th percentile (upper quintile) of these values. Areas with estimated prevalence above this threshold were classified as high prevalence areas, representing locations where prevalence exceeded that of 80^th^ percentile of all other areas in the country. More details on this subsection are provided in the supplementary materials (S1 File).

### Identification of high-risk areas

We used a generalized additive model (GAM) with location based smoothing function to estimate risk of overweight and obesity at local scales across the country. To account for the overestimation of the odds ratio effect measure when the outcome of interest is common (i.e., prevalence >10%) [45], the model was constructed using a modified Poisson regression which includes the log link function and Poisson family with robust variance-covariance estimator [46–50]. Given that the data is from cross-sectional studies, and that the overall prevalence of overweight and obesity is greater than 10%, the model was fitted to estimate the prevalence ratios (PR) [51]. We first fitted an unadjusted (crude) spatial model that included only latitude and longitude to capture the baseline spatial pattern of the risk of overweight and obesity. We then assessed how individual factors modified this spatial pattern (factor’s contribution) by extending the unadjusted model to include each risk factor separately, and finally all factors simultaneously. More details on the methodology are provided in the supplementary materials (S1 File).

### Ethical approval

We used secondary survey data that is publicly available and accessible from the DHS program online data repository and does not require ethical clearance for secondary analysis. Procedures and survey protocols for the surveys were reviewed by the ICF IRB and local IRB to ensure the survey complies with the U.S. Department of Health and Human Services regulations for the protection of human subjects (45 CFR 46) and local norms as explained in detail through https://www.dhsprogram.com/methodology/Protecting-the-Privacy-of-DHS-Survey-Respondents.cfm. All adult respondents gave informed consent and the data does not contain information that can identify individual participants.

## Results

### Descriptive statistics

A total of 33,787 (9,029 for 2010, 11,940 for 2015−16, and 12,818 for 2022 surveys) participants aged between 15–49 years were included in the analysis (Fig 1). The mean age was 29 (SD = 10.00) years both in females and males and for all surveys. The distribution of participants across socio-demographic factors is presented in the supplementary Table (S2 Table). In terms of residence, 70% of women lived in rural areas in 2010, 63% in 2015−16, and 64% in 2022. Among men, 66% resided in rural areas in 2022. Regarding marital status, 51% of men were in marital unions in 2022, while the proportion of women in marital union declined from 61% in 2010 to 58% in 2022. Contraceptive use among women increased from 32% in 2010 to 36% in 2015−16 before decreasing to 34% in 2022. In terms of education level, 90% of men had primary education or higher in 2022. For women, 78% had primary or higher education in 2010, 86% in 2015−16, and 84% in 2022. The percentage of women without work increased from 21% in 2010 to 36% in 2022 and the percentage of women in the middle or above wealth quintile was 65% in 2010, 68% in 2015−16, and 68% in 2022. The proportion of women aged 20 years or older remained relatively stable (78% in 2010 and 2015−16, and 79% in 2022), while 75% of men were aged 20 or older in 2022.

### Trends in observed prevalence of overweight and obesity

Over the past ten years, prevalence of overweight and obesity increased from 22% in 2010 to 32% in 2022 in women (Table 1). The prevalence increased with age, peaking in the 40–49 age group across all surveys, and was higher in urban areas compared to rural areas. Women with higher education and those who were wealthier had highest prevalence, with the richest reaching 51% in 2022, compared to 14.79% in the poorest in the same year. Contraceptive users had higher prevalence than non-users among women, with the gap widening over time from 31% in 2010 to 39% in 2022. Comparing women and men in 2022 (Table 1), the prevalence of overweight and obesity was higher in females (32%) than in males (15%) and increased with age, from 12% among females and 4% among males aged 15–19–48% and 26% in those aged 40–49, respectively. Urban residents had higher prevalence compared to rural residents, with 45% of urban females and 23% of urban males affected. The prevalence also increased with wealth index and education levels in both sexes. Respondents in the richest class had a prevalence of 51% in females and 28% in males, compared to 15% and 8% in females and males, respectively in the poorest class. Individuals with higher education had the highest prevalence of overweight and obesity (61% for females and 36% for males) and those with work which does not involve physical or manual activities had the highest prevalence (52% in females and 26% in males), while those without occupations had the lowest prevalence.

**Table 1:** Prevalence of overweight or obesity by socio-demographic factors.

Factor	2010		2015−16		2022			
	Female		Male		Female		Male	
	Yes (%)	N (Weighted)	Yes (%)	N (Weighted)	Yes (%)	N (Weighted)	Yes (%)	N (Weighted)
Age group^a^								
15–19	207(10.38)	1992	284(10.81)	2632	175(11.82)	1480	61(4.26)	1444
20–29	631(20.72)	3042	990(24.86)	3981	627(26.72)	2348	194(10.89)	1784
30–39	723(29.87)	2421	1163(38.18)	3045	745(41.32)	1804	313(21.44)	1458
40–49	481(29.39)	1638	959(41.96)	2286	678(47.60)	1423	274(25.5)	1076
Residence^a^								
Urban	1004(37.27)	2693	1830(41.62)	4397	1133(44.82)	2529	437(22.56)	1938
Rural	1038(16.22)	6400	1566(20.75)	7548	1091(24.12)	4525	406(10.60)	3825
Marital Status^a^								
Never	355(14.48)	2454	578(17.99)	3214	432(21.11)	2044	179(7.13)	2517
In union	1387(25.1)	5527	2301(32.28)	7127	1450(35.47)	4087	619(21.07)	2937
Separated	299(26.92)	1111	517(32.25)	1604	344(37.23)	924	45(14.42)	309
Education^a^								
None	295(17.66)	1670	361(21.1)	1709	264(23.87)	1107	58(10.19)	574
Primary	1285(21.98)	5843	2063(28.01)	7365	1222(32.38)	3774	442(14.12)	3134
Secondary	430(27.96)	1537	871(32.17)	2706	687(32.89)	2090	272(14.63)	1858
Higher	32(77.09)	42	103(62.24)	165	51(61.23)	84	70(35.51)	197
Occupation^a^								
None	335(17.89)	1875	619(22.37)	2768	623(24.35)	2557	46(5.34)	871
Not Manual	139(61.88)	225	257(56.41)	456	527(51.83)	1017	199(26.31)	758
Manual	1567(22.41)	6994	2520(28.9)	8721	922(29.27)	3153	547(14.26)	3834
Other	–	–	–	–	152(46.46)	328	50(16.72)	300
Wealth index^a^								
Poorest	134(9.02)	1490	232(11.94)	1939	153(14.79)	1037	68(7.74)	883
Poorer	199(11.82)	1683	317(15.82)	2005	260(21.32)	1220	77(7.40)	1037
Middle	263(15.11)	1740	434(20.59)	2107	352(25.03)	1405	111(9.32)	1191
Richer	494(25.63)	1928	851(33.13)	2568	543(34.39)	1579	220(16.21)	1355
Richest	951(42.25)	2252	1563(47.00)	3325	917(50.55)	1813	367(28.29)	1298
Contraceptive use^a^^,^^b^								
Yes	893(30.62)	2917	1562(36.52)	4276	928(39.01)	2377	N/A	N/A
No	1149(18.6)	6176	1835(23.92)	7669	1297(27.74)	4677		
Total (weighted)	**2042(22.46)**	**9093**	**3396(28.43)**	**12000**	**2225(31.54)**	**7054**	**843(14.62)**	**5763**

^a^Indicate that the difference between groups/categories is statistically significant (p < 0.05).

^b^Contraceptive use data were only available for women.

**Fig 2:**
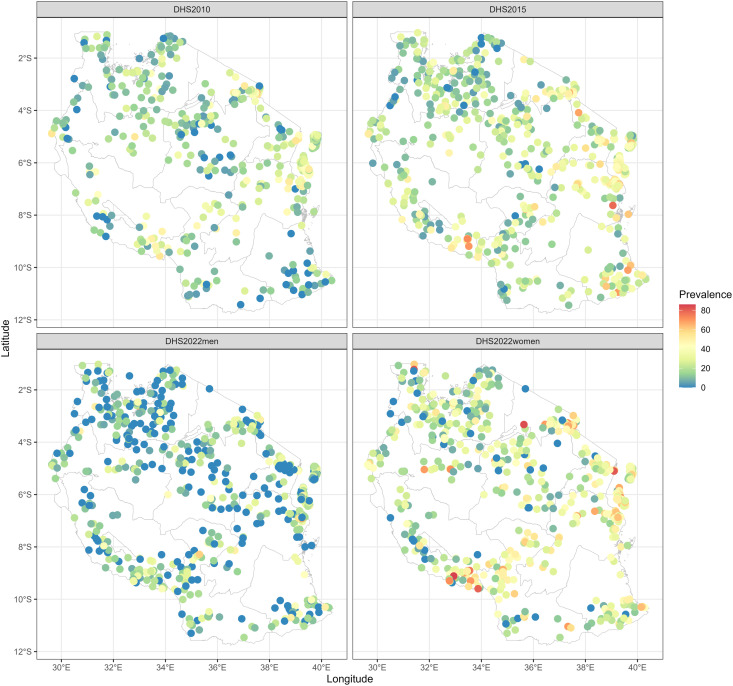
Distribution of observed prevalence of overweight or obesity in Tanzania for women in 2010, 2015, and 2022, and for Men in 2022. Each circle represents a sampled and georeferenced cluster. The maps were created by the authors using R version 4.3.3. The shapefile was sourced from Natural Earth Data (https://www.naturalearthdata.com/), which is available under a public domain license (https://www.naturalearthdata.com/about/terms-of-use/), and was accessed through the *rnaturalearth* R package.

Fig 2 shows the sampled clusters and their corresponding observed prevalence of overweight and obesity for women and men. It is evident that prevalence increased over time and that the distribution is inhomogeneous across the country. Prevalence was higher along the eastern part of the country, southern highlands, and along the northern part of the country. However, there were also clusters of high prevalence in other parts of the country. In contrast for men, few clusters of high prevalence were observed in men, particularly along the eastern and southern highlands zones.

### Spatial and temporal trends in subnational prevalence of overweight and obesity

**Fig 3:**
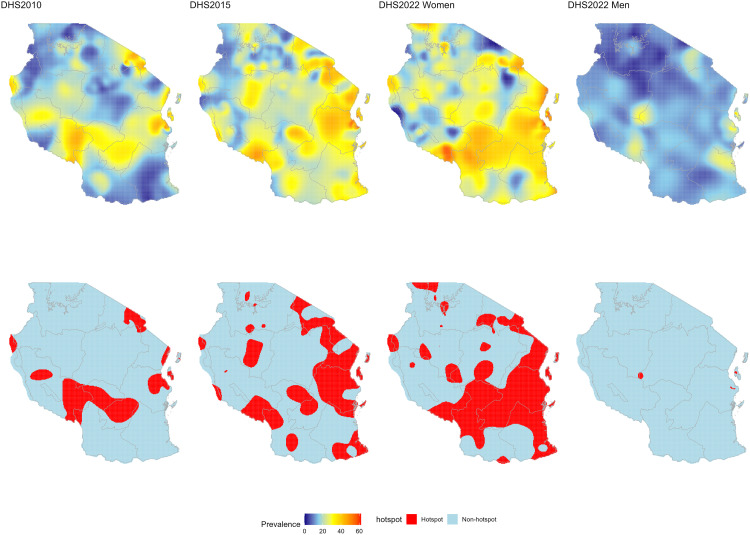
Spatial and temporal trends in subnational estimates of prevalence of overweight or obesity in peoples aged 15-49 years. Top row: Interpolated prevalence of overweight and obesity. Bottom row: Areas of high prevalence (≥ 80^th^ percentile or top quintile, equivalent to prevalence ≥ 29.39% or hotspot). Columns 1 to 3 present prevalence and areas of high prevalence estimates for women and column 4 present estimates for men. These maps were created by the authors in R (version 4.3.3) using a shapefile from Natural Earth Data (https://www.naturalearthdata.com/) accessed via the *rnaturalearth* package.

Fig 3 presents the prevalence surface maps of overweight and obesity among women and men (row 1), along with the identified high prevalence areas (defined as areas with prevalence ≥ 80^th^ percentile, equivalent to prevalence ≥ 29.39%) across the three surveys (row 2). The prevalence of overweight and obesity in women was unevenly distributed across the country and increased over time. In 2010, overweight and obesity was predominantly low, with most areas showing low prevalence and only a few localized areas in the eastern, south-western highlands, and northern zones exhibiting high prevalence levels. In 2015−16, there was an expansion of prevalence, with more areas shifting towards higher prevalence levels. In 2022, prevalence increased significantly, with many areas transitioning to higher prevalence levels. The highest prevalence in women appeared to be concentrated in the Eastern zone, Zanzibar, Njombe and Iringa in Southern highlands, Mbeya and Songwe in the South western highlands, eastern part of the Southern zone, and the Northern zone. Similar pockets of high prevalence were found to emerge in the Central, Western, and Lake zone. It was also found that since 2010, the geographic spread of areas with high prevalence (areas in the upper quintile or prevalence ≥ 80th percentile) among women has been expanding across the country (row 2, columns 1–3). Unlike for women, prevalence in men was low compared to that of women in 2022, exhibited a different spatial pattern, with relatively high prevalence in some of regions compared to other areas. These regions include Lindi in the Southern zone, Dar es Salaam and Unguja in the eastern zone, Mbeya in the South western highlands, Arusha in the Northern zone, and along the border areas of the Central, Western, and South western highlands zones (column 4, rows 1 and 2).

### Factors and spatial distribution of the risk of overweight and obesity

Table 2 presents results of adjusted prevalence ratios for overweight and obesity based on the GAMs model. The risk of overweight and obesity was influenced by multiple socio-economic and demographic factors, with significant differences observed between women and men and across years. Age was associated with higher prevalence ratios of overweight and obesity, with women aged 40–49 years having 2.38 (95% CI: 1.92–2.96) in 2010, 3.34 (95% CI: 2.82–3.95) in 2015−16, and 3.49 (95% CI: 2.86–4.26) in 2022 prevalence ratios than those aged 15−19 years. For men, those aged 40−49 years had 3 (2.98; 95% CI: 2.08–4.27) times higher prevalence ratio of overweight/obesity than those aged 15−19 years in 2022. Wealth index was associated with overweight and obesity, with the richest women having 3 times higher PRs than poor women in each survey (PR: 3.32; 95% CI: 2.68–4.11) in 2010, 3.30 (95% CI: 2.80–3.90) in 2015−16, and 2.58(95% CI: 2.10–3.16) in 2022, respectively). Likewise, richest men had 3 times higher prevalence ratio (3.29; 95% CI: 2.40–4.52) compared to the poorest in 2022. Urban residence was significantly associated with higher PRs for women in 2010 (1.18; CI: 1.05–1.32) and 2015−16 (1.10; 95% CI: 1.01–1.20), but this association weakened in 2022 and was not significant for men. In addition, women in union had 36% and 29% higher PRs thank those not in union in 2010 (1.36; 95% CI: 1.15–1.60) and 2015−16 (1.29; 95% CI: 1.15–1.46), respectively, though this was not significant in 2022. Whereas men in union had 69% higher PR (1.69; 95% CI: 1.34–2.12) than those not in union in 2022. Education showed mixed associations, with secondary education among women being significant in 2015−16, having 17% higher PR (1.17; 95% CI: 1.03–1.34) than those who did not go to school. Non-manual workers had a 32% (1.32; 95% CI: 1.06–1.64) higher PR than their counterparts in 2010 for women and 46% (1.46; 95% CI: 1.04–2.05) higher PR for men in 2022. Finally, contraceptive users in women had 10% (1.10; 95% CI: 1.00–1.22) and 14% (1.14; 95% CI: 1.06–1.22) higher PRs in 2010 and 2015−16, respectively, but this association was not statistically significant in 2022.

**Table 2:** Adjusted prevalence ratios of overweight and obesity to show association between the outcome and factors.

Factor	2010			2015−16		2022			
	Women			Women		Women		Men	
	APR(CI)	P-value	APR (CI)		P-value	APR (CI)	P-value	APR (CI)	P-value
**Age group**									
15 - 19	Ref		Ref			Ref		Ref	
20 - 29	1.56(1.30-1.89)	<0.001	1.93(1.66-2.24)		<0.001	1.86(1.56-2.23)	<0.001	1.69(1.24-2.30)	0.001
30 - 39	2.18(1.77-2.69)	<0.001	2.85(2.42-3.35)		<0.001	2.99(2.46-3.63)	<0.001	2.46(1.73-3.48)	<0.001
40 - 49	2.38(1.92-2.96)	<0.001	3.34(2.82-3.95)		<0.001	3.49(2.86-4.26)	<0.001	2.98(2.08-4.27)	<0.001
**Residence**									
Rural	Ref		Ref			Ref		Ref	
Urban	1.18(1.05-1.32)	0.007	1.10(1.01-1.20(0.79-1.16)		0.028	1.10(1.00-1.22)	0.060	0.98(0.83-1.15)	0.773
**Marital Status**									
Never	Ref		Ref			Ref		Ref	
In union	1.36(1.15-1.6)	<0.001	1.29(1.15-1.46)		<0.001	1.12(0.95-1.29)	0.080	1.69(1.34-2.12)	<0.001
Separated	1.37(1.12-1.67)	0.002	1.21(1.04-1.40)		0.011	1.12(0.95-1.32)	0.176	1.30(0.92-1.83)	0.143
**Education**									
None	Ref		Ref			Ref		Ref	
Primary	0.99(0.86-1.14)	0.870	1.11(0.99-1.25)		0.070	1.09(0.96-1.25)	0.187	0.91(0.71-1.18)	0.489
Secondary	1.06(0.90-1.26)	0.478	1.17(1.03-1.34)		0.020	1.16(0.99-1.35)	0.061	1.01(0.76-1.34)	0.959
Higher	1.34(0.88-2.04)	0.172	1.21(0.90-1.61)		0.202	1.20(0.88-1.66)	0.253	1.26(0.87-1.84)	0.223
**Occupation**									
None	Ref		Ref			Ref		Ref	
Not manual	1.32(1.06-1.64)	0.012	1.17(0.99-1.37)		0.063	1.13(1.00-1.28)	0.056	1.46(1.04-2.05)	0.027
Manual	1.05(0.93-1.19)	0.427	1.06(0.97-1.17)		0.194	0.99(0.89-1.09)	0.813	1.26(0.92-1.73)	0.146
Other	–	–	–		–	1.08(0.91-1.29)	0.390	1.33(0.88-2.02)	0.181
**Wealth index**									
Poorest	Ref		Ref			Ref		Ref	
Poorer	1.18(0.95-1.47)	<0.144	1.34(1.13-1.59)		0.001	1.44(1.18-1.75)	<0.000	1.00(0.72-1.40)	0.979
Middle	1.50(1.22-1.85)	<0.001	1.63(1.39-1.92)		<0.001	1.56(1.29-1.89)	<0.001	1.40(1.03-1.89)	0.029
Richer	2.30(1.89-2.80)	<0.001	2.52(2.16-2.94)		<0.001	2.00(1.65-2.43)	<0.001	2.04(1.52-2.74)	<0.001
Richest	3.32(2.68-4.11)	<0.001	3.30(2.80-3.90)		<0.001	2.58(2.10-3.16)	<0.001	3.29(2.40-4.52)	<0.001
**Contraceptive use** ^b^									
No	Ref		Ref			Ref			
Yes	1.10(1.00-1.22)	0.046	1.14(1.06-1.22)		0.001	1.09(1.00-1.19)	0.056	–	–

^b^Contraceptive use data were only available for women.

**Fig 4:**
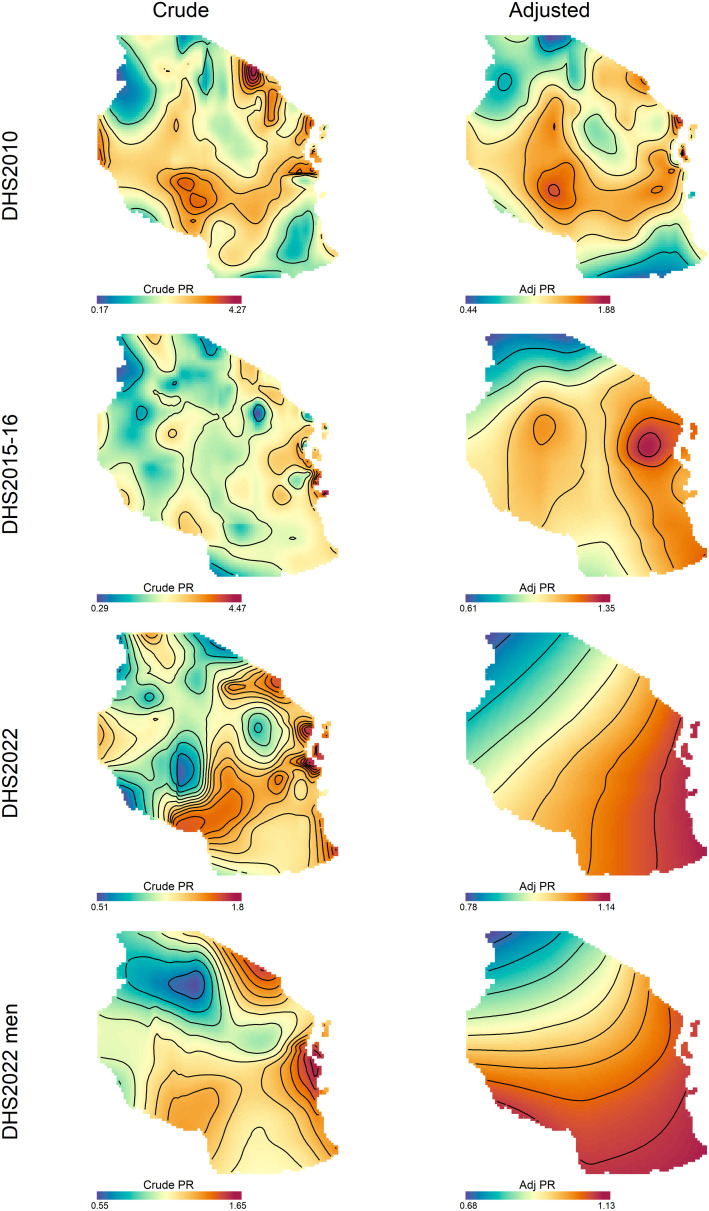
Spatial distribution of crude(unadjusted) and adjusted prevalence ratios of overweight or obesity for women in DHS 2010 (Row 1), DHS 2015−16 (Row 2), and DHS 2022 (Row 3), and for men in DHS 2022 (Row 4). Contour lines indicate areas of significantly increased or decreased prevalence ratios. These maps were created by the authors in R (version 4.3.3) using a shapefile from Natural Earth Data (https://www.naturalearthdata.com/) accessed via the *rnaturalearth* package.

Fig 4 presents spatial and temporal patterns of risk of overweight and obesity across Tanzania, comparing between crude and adjusted prevalence ratios. In 2010, high risk areas based on crude prevalence ratios were predominantly concentrated in the Northern zone, South west highlands, parts of Southern highlands, the Eastern zone, and the Western zone. By 2015−16, these high risk areas shifted slightly, clustering along the eastern coast of the Indian Ocean, with Mbeya and Kilimanjaro continuing to exhibit relatively high crude prevalence ratios. In 2022, high risk areas clustered on the Eastern coast, Northern, Southern, Southern highlands, and part of South western highlands. For men in 2022, the Eastern zone and Zanzibar showed the highest risk of overweight and obesity. Other parts of the country with higher risk included the Northern zone, Southern zone, Southern highlands, and South west highlands. Higher risks were observed in women compared to men, with most parts of the Lake zone, western regions, and the central areas showing lower risk for men compared to women. Adjusting for all risk factors smoothed the high risk areas, maintaining a similar overall risk distribution but showing reduced risk across all surveys and for both sexes. While crude prevalence ratio maps showed greater variability, with extreme prevalence ratios for women of up to 4.27, 4.47, and 1.8 in 2010, 2015−16, and 2022 respectively, adjusting for factors reduce this variability to a maximum of 1.88, 1.35, and 1.14 for women in 2010, 2015−16, and 2022 respectively. Similarly, crude prevalence ratios for men in 2022 ranged from 0.55 to 1.65, while adjusted prevalence ratios ranged from 0.68 to 1.13.

**Fig 5:**
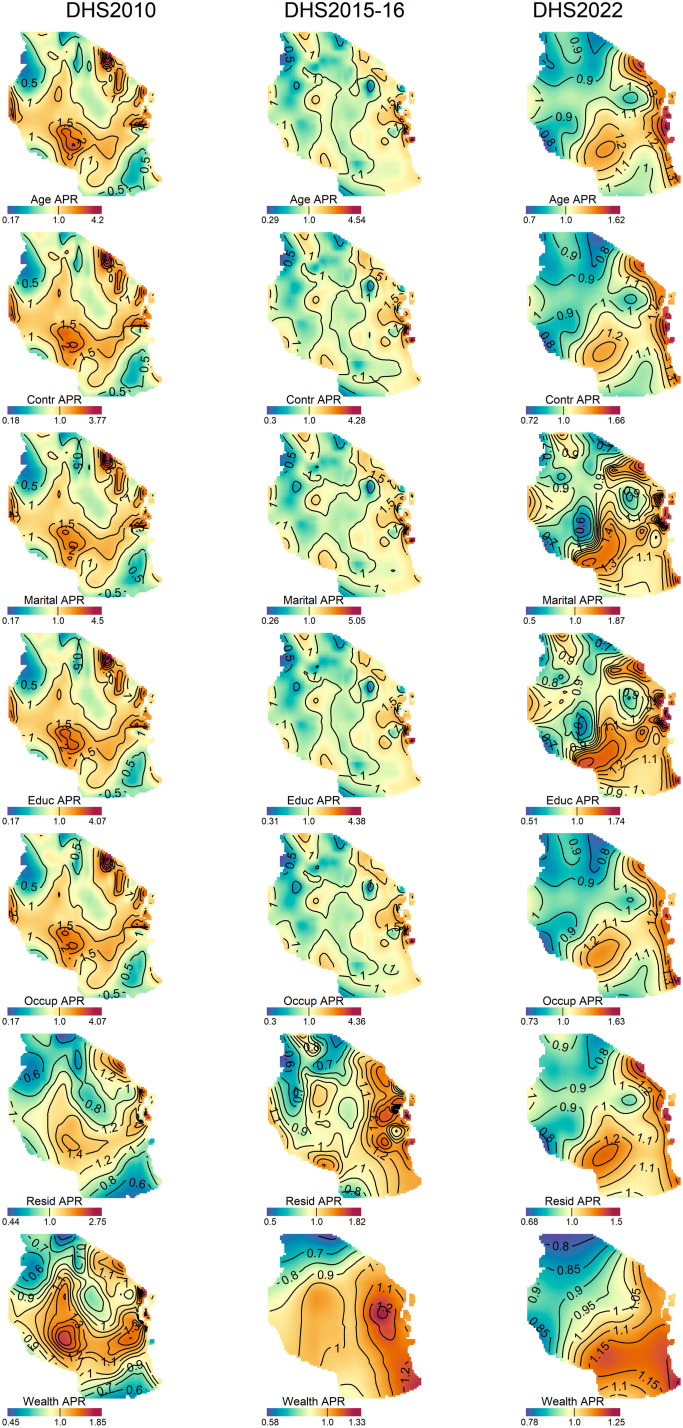
Contribution of each risk factor to the spatial distribution of the risk of overweight or obesity in women aged between 15 and 49 years for DHS 2010 (column 1), DHS 2015−16(column 2), and DHS 2022 (column 3). Contour line indicates areas of significantly increased or decreased prevalence ratios. These maps were created by the authors in R (version 4.3.3) using a shapefile from Natural Earth Data (https://www.naturalearthdata.com/) accessed via the *rnaturalearth* package.

**Fig 6:**
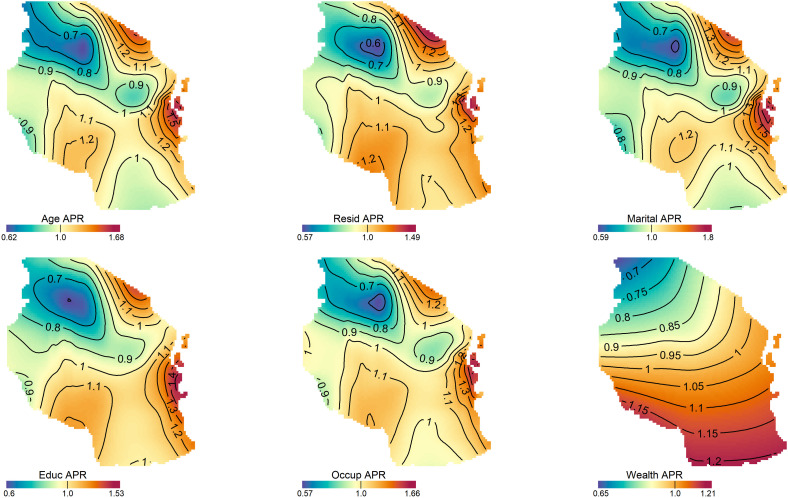
Contribution of each risk factor to the spatial distribution of overweight and obesity in men aged between 15 and 49 years for DHS 2022. Contour line indicates areas of significantly increased or decreased prevalence ratios. These maps were created by the authors in R (version 4.3.3) using a shapefile from Natural Earth Data (https://www.naturalearthdata.com/) accessed via the *rnaturalearth* R package.

In women, the contribution of each factor to the spatial distribution of the risk of overweight and obesity show notable temporal and regional variations across surveys and risk factors (Fig 5, S3 Table). Age demonstrated substantial geographic disparities over the study period. In 2010, the PRs for age ranged from 0.17 to 4.27, which widened to 0.29–4.54 in 2015–16 but narrowed considerably to 0.7–1.62 by 2022, reflecting a reduction in spatial variability. Similarly, marital status exhibited a broad range of PRs in 2010 (0.17–4.5), which persisted in 2015–16 (0.26–5.05) but showed a marked decrease in disparities by 2022 (0.5–1.87). Contraceptive use followed a similar trend, with a decline in PR variability over time, narrowing from 0.18–3.77 in 2010 to 0.72–1.66 in 2022. Education, occupation, residence, and wealth index also exhibited significant geographic heterogeneity in earlier years, which diminished by 2022. The PRs for education ranged from 0.17–4.07 in 2010 to 0.51–1.74 in 2022 and occupation-related disparities narrowed from 0.17–4.07 in 2010 to 0.73–1.63 in 2022. Residence showed a similar pattern, with PRs reducing from 0.44–2.75 in 2010 to 0.68–1.5 in 2022 while wealth index demonstrated progressive reductions in spatial disparities over time, with PRs narrowing from 0.45–1.85 in 2010 to 0.78–1.25 in 2022. For men (Fig 6, S3 Table), the maximum values of PRs for age, occupation, and marital status were relatively higher than crude prevalence ratios. The spatial risk pattern following adjustment for age and marital status also differed considerably from those of the crude PRs. In contrast, the spatial patterns of PRs for residence, education, and wealth index were relatively similar to that of the crude PRs. Notably, the pattern and range of PRs for wealth index closely resembled those of the adjusted PRs for all factors.

## Discussion

In this study, we estimated prevalence of overweight and obesity, identified high-risk areas, and assessed the contribution of socio-demographic factors on the spatial distribution and temporal trends of overweight and obesity in Tanzania. Between 2010 and 2022, the prevalence of overweight and obesity in women increased by 45.45%, rising from 22% in 2010 to 32% in 2022. In 2022, the prevalence among men was 15%, lower than that of women in the same year. Moreover, overweight and obesity was strongly associated with age, wealth, and place of residence. This association was stronger in women, particularly those in older age groups and higher economic status. In terms of geographical disparities, the Lake zone consistently showed a lower risk, while markedly higher prevalence rates were observed in the Eastern, Northern, Southern, South western, and Southern highland zones. Further analysis indicated that socio-economic and demographic factors reduced overall risk levels and smoothed spatial risk areas, while preserving the broader geographic risk pattern, highlighting their role in shaping overweight and obesity disparities across the country.

The rising prevalence of overweight and obesity observed in this study aligns with findings from other studies, which have documented a substantial increase in overweight and obesity among adults and children over the past decades [9,10]. Since 1990, the prevalence has doubled, and projections suggest that over half of the global adult population may become overweight or obese by 2050 [9,52]. This trend is evident across both developed and developing countries, driven by factors such as economic growth, urbanization, and dietary shifts toward processed and high-calorie or starchy foods [53,54]. Regions like the Americas and Europe report the highest obesity rates, while low and middle-income countries are experiencing a rapid increase due to the ongoing nutrition transition [9,10,55].

The gender disparities in prevalence between women and men, particularly in 2022 can be attributed to a combination of socio-economic factors, socio-cultural norms, and biological factors. In many African cultures, a larger body size, particularly on the hip in women is associated with beauty and social status [56], while gender roles sometimes influence dietary habits and may limit participation in physical activities, further contributing to higher burden of overweight and obesity [57]. Hormonal fluctuations experienced by women during key life stages also play a role in weight gain [58]. Together, these aspects foster behaviours and conditions that increase the risk of overweight and obesity, necessitating gender-sensitive strategies be implemented to address the growing burden of overweight and obesity. Furthermore, our findings indicate that women who used contraceptives had a higher prevalence of overweight and obesity. This association is consistent with previous studies reporting weight gain as a potential side effect of hormonal contraceptive [41,59]. Studies indicate that the underlying mechanism may involve hormonal changes that influence appetite and metabolism [60].

Spatial disparities in overweight and obesity prevalence across the country highlight existing differences in social, economic, demographic, and nutritional factors. However, over time, these disparities have narrowed significantly, with reduced geographic variability observed in 2022. This expansion suggests that overweight and obesity are no longer confined to specific regions or urban centers but are becoming more prevalent across diverse geographic and demographic settings. The high prevalence areas identified in this study were consistent with the location of geographical clusters identified in other studies using similar methods [19]. However, the continuous surface prevalence maps and the prevalence ≥ 80^th^ percentile approach used in this study revealed more detailed spatial structures. These spatial patterns reflect underlying shifts in socioeconomic status, urbanization, lifestyle behaviours, and dietary transitions occurring across the country.

Our findings show that adjusting for socio-economic and demographic factors narrowed the range of prevalence ratios, illustrating the epidemiological importance of these factors in shaping risk patterns. The observed and estimated prevalence distributions, along with the changing spatial trends reflect the country’s ongoing socio-economic progress and demographic transitions and their implication on the current spatial and temporal patterns of overweight and obesity. The findings show that overweight and obesity are influenced by a range of socio-demographic factors, including age, wealth, and place of residence. Among these, wealth index and age had the strongest effects on prevalence ratios and both wealth and residence contributed to a narrowing of the gap between the minimum and maximum values. Evidence from Tanzania [61], and various countries in Asia demonstrate that higher income is associated with increased age-standardized prevalence of overweight and obesity [62–66]. Similar trends have been observed in other sub-Saharan African countries, where women in wealthier quintiles had greater prevalence of overweight and obesity [63,67,68]. Individuals with higher economic status often reside in urban environments where easy access to starchy foods and sedentary lifestyles promotes overconsumption of these foods and reduces physical activity [17,69]. Interestingly, this trend contrasts with patterns observed in high-income countries, where overweight and obesity are more prevalent among individuals with lower socioeconomic status [70,71]. It’s a striking reminder of how the drivers of health can shift dramatically depending on the socio-economic and cultural context.

With the increasing burden of non-communicable diseases, this study has provided valuable information into one of their modifiable NCD risk factors. Numerous studies have demonstrated that overweight and obesity significantly increase the risk of developing NCDs [1–4]. The GBD studies have consistently ranked high BMI as one of the leading risk factors across multiple GBD iterations [72], with its burden increasing in many countries both in adults and children [73–75], and contributing to the burden of NCDs. Our results highlight the need to prioritize overweight and obesity in NCDs prevention efforts and implementing comprehensive strategies to reverse this glowing health issue in the country. These should include education programs that promote behaviour change, lifestyle modification, good nutrition, and development of infrastructures that encourage physical activities. Such integrated interventions are critical in reversing the escalating trend of overweight and obesity in the country.

This study has a number of limitations to be noted. First, the use of cross-sectional data limits our ability to establish causal relationships between predictors and overweight/obesity. Additionally, the analysis was limited to individuals aged 15–49 year and included a relatively limited number of socio-demographic factors, without explicitly capturing nutritional or dietary factors, eating habits and physical activity which play a significant role in weight gain and may interact with other predictors. Data on dietary diversity and consumption of unhealthy foods and beverages were only available for women in the 2022 survey, and were not collected for men or in the 2010 and 2015–16 surveys. As a result, these key dietary indicators could not be included in the analysis. Likewise, physical activity was collected to measure shortness of breath in the 2022 survey and does not adequately capture habitual or long-term physical activity patterns to be included in this analysis. The exclusion of these aspects limits the ability of the study to capture full spectrum of factors influencing overweight and obesity. Furthermore, we were unable to assess the trend in overweight and obesity among men, as only one survey round included male participants. While we accounted for complex survey design by applying sampling weights, stratification, and clustering which provides valid population level estimates and adjusts standard errors, this approach does not explicitly model regional or zonal variations. As such, unobserved heterogeneity at higher administrative levels may not be fully captured. Multilevel models could offer a complementary approach to better account for hierarchical structure of the data. Despite these limitations, the data used in this study are country representative, enhancing the generalizability and validity of its findings. Additionally, the study provided important information in understanding the spatial-temporal trends and regional disparities in overweight and obesity across Tanzania and identified high-risk areas, providing a foundation for monitoring and targeting interventions in regions with increased risk.

## Conclusions

The prevalence of overweight and obesity is high among women than men and is on the increasing trajectory, with wealth, age, and place of residence identified as the most influential socio-demographic factors. Significant within-country disparities were observed, with Eastern, Northern, Southern, South western, and Southern highland zones presenting higher risks than other zone. These findings are crucial and highlight the urgent need for early interventions to address the growing burden of overweight and obesity, particularly in the context of shifting lifestyles and increasing urbanization in Tanzania. Efforts should prioritize high risk demographic groups while also adapting to the changing geographic patterns of overweight and obesity. As overweight and obesity become more homogeneously distributed across the country, comprehensive and population-wide strategies will be essential to reverse this growing health challenge.

## Supporting information

S1 File Supplementary methods. (DOCX)

S2 Table Distribution of participants across socio-demographic factors. (DOCX)

S3 Table Comparisons of unadjusted and adjusted variable effects on overweight and obesity. (DOCX)
