# Supplementary material for: Trends and geographic patterns of overweight and obesity among Tanzanian adults: Evidence from the 2010–2022 Demographic and Health Surveys
Source: PLoS One. 2025 Sep 22;20(9):e0332275. doi: 10.1371/journal.pone.0332275 (PMC12453257; doi:10.1371/journal.pone.0332275)
Supplement: S1 File — (DOCX) [file pone.0332275.s001.docx]

**Supplemental methods to “**Trends and geographic patterns of overweight and obesity among Tanzanian adults: evidence from the 2010–2022 Demographic and Health Surveys”

**Subnational prevalence estimation**

Health metrics are mostly spatially related and exhibit dependence due to influence by various factors. To account for these dependencies, interpolation methods were applied to predict health metrics at unsampled locations using data from sampled locations [1,2]. By considering spatial relationships between areas, these methods offer valuable insights into the health status of populations in areas lacking direct observations. To obtain subnational estimates of prevalence of overweight and obesity across the country, we used the Kernel density estimation (KDE), an interpolation method that uses features within a neighbourhood to estimate values at locations with unmeasured prevalence. Following [3], mathematically, we estimated the intensity surface*,* $z\left( x,y \right)$ according to the following formula:

$$z\left( x,y \right)= \sum_{i=1}^{n} \frac{1}{h_{i}^{2}}K\left( \frac{d_{i}}{h_{i}} \right)$$

where, $n$ is the number of observed overweight and obesity cases, $d_{i}$ is the geometrical distance between each observed case at each location (*x,y)*, $K$ is the kernel density function, and $h_{i}$ is the bandwidth used for each case *i*. Given that our data was georeferenced at cluster level, we used *PrevR* package in R [3] which uses Gaussian kernel estimators with an adaptive bandwidth of equal number of observations to interpolate prevalence surfaces. This kernel density estimation uses adaptive bandwidths based on a minimal number of observations (*N*) obtained through the *Noptim()* function within *PrevR* [4]. *N* is dependent on the observed national prevalence, number of people sampled, and surveyed and geolocated clusters. The surface is a weighted estimate of overweight and obesity prevalence surface with parameter *N* for each survey separately, and men and women, according to our dataset. This method has been used elsewhere in health research [4–6]. For comparison purposes, we estimated areas of high prevalence of overweight and obesity by combining the continuous surface prevalence data, and computed the combined upper quintile (80^th^ percentile). This enabled identification of high prevalence areas and comparison across surveys.

**Identification of high-risk areas using Generalized Additive Models**

We used generalized additive models (GAMs) which allows for location based smoothing functions to estimate risk of overweight and obesity at local scales. Given that respondents belong to either

$$Y\left( x,y \right)=\left\{ \begin{aligned} 1, \text{if respondents have overweight or obesity} \\ 0, &\text{Otherwise} \end{aligned} \right.$$

at each location $(x,y)$, then, $Y\left( x,y \right)$ follows a Bernoulli distribution from a binomial family with $p\left( x,y \right)$ being the probability of having overweight or obesity and $1- p\left( x,y \right)$ the probability that the respondent at location $\left( x,y \right)$ does not have overweight or obesity. However, to account for the overestimation of the effect measure when the outcome of interest is common (i.e. prevalence >10%) [7], the model was constructed using a modified Poisson regression which includes the log link function and Poisson family with robust variance-covariance estimator [1,2,8–10]. Because the data used in this study is from cross-sectional studies and that the overall prevalence of overweight and obesity is greater than 10%, the GAMs were fitted to estimate the prevalence ratios (PR) [11] at every location in the country. These models are part of the non-parametric or semi-parametric regression models allowing to model the spatial effects of the geographical locations of binary outcomes using a log link and Poisson family with robust variance-covariance estimator while adjusting for socio-demographic factors. Mathematically [12,13], the GAM model is given by:

$$log[p\left( x,y \right)]=\beta_{0}+\beta X_{\left( x,y \right)}+S(x,y)$$

where $p$ is the probability of having overweight or obesity, $\beta_{0}$ is the intercept, $\beta$ is a vector of coefficients of adjustment socio-demographic risk factors $X_{\left( x,y \right)}$. $log[p\left( x,y \right)]$ is a log link function representing the log of prevalence ratio of overweight or obesity at location $\left( x,y \right)$ with the prevalence ratios being given by $exp\left( log[p\left( x,y \right)] \right)$. $S(x,y)$ is a 2-dimensional non-parametric smoothing nonlinear function for modelling location of respondents, and $(x,y)$ represent latitudes and longitudes. In the absence of the smoothing function, $S(x,y)$, the model becomes an ordinary logistic regression, and when the socio-demographic factors $X_{\left( x,y \right)}$ are ignored, the model produces unadjusted results.

The above model was implemented in R through the *MapGAM* package [14] to generate predictive maps of prevalence ratios of overweight and obesity across the country in the absence (unadjusted) or presence of risk factors. A spatial prediction grid was generated based on the cluster coordinates available in the dataset for each survey and the country boundary polygon (obtained from Malaria Atlas using the *MalariaAtlas* package in R) using the *predgrid* function. The *modgam* function was used for fitting the models with the log link function and Poisson family with robust variance estimator to estimate the prevalence ratios across all grid points. Smoothing depends on span size and a lowess smoother [15,16], thus, adapting to changes in population density. Here, the span size is the percentage of the data points in the neighbourhood. Loess, a locally-weighted regression smoother, was used to adjust neighbourhood size based on local population density while preserving smoothness. It uses the k-nearest neighbours and weights them with a tricube distance function, giving more weight to points that are closer and zero weight to those that are far away [16]. We determined the optimal amount of smoothing for model by minimizing the Akaike Information Criterion (AIC). The *optspan()* function in *MapGAM* was used to find an optimal span size for the loess smoother [15,16] and tested statistical significancy of global spatial effect in all the models. This method has been used elsewhere in public health [17–19]. In line with the DHS Guide to Statistics [20], we accounted for the complex survey design by employing a weighted analysis approach using the “survey” package in R [21].

**Risk areas identification using Generalized additive models**

We used the Generalized Additive Models (GAMs) with location-based smoothing functions to estimate the spatial distribution of overweight and obesity risk across the country. These models were implemented in R using the *MapGAM* package, which allows the incorporation of smooth functions of geographic coordinates (latitude and longitude) to model spatial variation. We first fitted an unadjusted spatial model that included only the geographic coordinates to capture the baseline risk spatial pattern of overweight and obesity. This served as the reference map. Next, to assess the contribution of each individual risk factor to the spatial variation, we added each risk factor separately into the model. To explore the extent to which specific factors explain spatial disparities in the risk of overweight and obesity and assess how each risk factor altered the spatial pattern, we compared the spatial distribution of prevalence ratios for each adjusted model to the unadjusted reference model.

**Subnational prevalence estimation and identification of high prevalence areas**

To obtain prevalence estimates of overweight and obesity to unsampled locations across the country, we used *PrevR* package [3] to interpolate the measured prevalence. This interpolation method uses features within a neighbourhood to estimate values at locations with unmeasured prevalence. This method has been used elsewhere in health research [4–6].

To assess how the spatial distribution of overweight and obesity changed over time, we identified areas with high prevalence of overweight and obesity by combining the continuous surface prevalence estimates derived from our models. To systematically identify high prevalence areas, we calculated the 80^th^ percentile (upper quintile) of the combined prevalence. This threshold was used to define areas where the estimated prevalence was higher than 80% of all other areas in the country.

**References**

1. Coutinho LMS, Scazufca M, Menezes PR. Methods for estimating prevalence ratios in cross-sectional studies. Rev Saúde Pública. 2008 Dec;42:992–8.

2. Cross-sectional studies | Revista de la Facultad de Medicina Humana [Internet]. [cited 2025 Jan 17]. Available from: http://revistas.urp.edu.pe/index.php/RFMH/article/view/3069

3. Larmarange J, Vallo R, Yaro S, Msellati P, Méda N. Methods for mapping regional trends of HIV prevalence from Demographic and Health Surveys (DHS). Cybergeo Eur J Geogr [Internet]. 2011 Oct 26 [cited 2024 Jun 21]; Available from: https://journals.openedition.org/cybergeo/24606

4. Larmarange J, Bendaud V. HIV estimates at second subnational level from national population-based surveys. AIDS Lond Engl. 2014 Nov;28(4):S469–76.

5. Keino BC, Carrel M. Spatial and temporal trends of overweight/obesity and tobacco use in East Africa: subnational insights into cardiovascular disease risk factors. Int J Health Geogr. 2023 Aug 24;22(1):20.

6. Nutor JJ, Duah HO, Agbadi P, Duodu PA, Gondwe KW. Spatial analysis of factors associated with HIV infection in Malawi: indicators for effective prevention. BMC Public Health. 2020 Jul 25;20(1):1167.

7. Lee J, Tan CS, Chia KS. A Practical Guide for Multivariate Analysis of Dichotomous Outcomes. Ann Acad Med Singapore. 2009 Aug 15;38(8):714–9.

8. Martinez BAF, Leotti VB, Silva G de S e, Nunes LN, Machado G, Corbellini LG. Odds Ratio or Prevalence Ratio? An Overview of Reported Statistical Methods and Appropriateness of Interpretations in Cross-sectional Studies with Dichotomous Outcomes in Veterinary Medicine. Front Vet Sci [Internet]. 2017 Nov 10 [cited 2025 Jan 17];4. Available from: https://www.frontiersin.org/journals/veterinary-science/articles/10.3389/fvets.2017.00193/full

9. Vega AC, Maguiña JL, Soto A, Lama-Valdivia J, López LEC. Estudios transversales: Cross-sectional studies. Rev Fac Med Humana. 2021;21(1).

10. Barros AJ, Hirakata VN. Alternatives for logistic regression in cross-sectional studies: an empirical comparison of models that directly estimate the prevalence ratio. BMC Med Res Methodol. 2003 Oct 20;3:21.

11. Gnardellis C, Notara V, Papadakaki M, Gialamas V, Chliaoutakis J. Overestimation of Relative Risk and Prevalence Ratio: Misuse of Logistic Modeling. Diagnostics. 2022 Nov 17;12(11):2851.

12. Zou G. A Modified Poisson Regression Approach to Prospective Studies with Binary Data. Am J Epidemiol. 2004 Apr 1;159(7):702–6.

13. Zou G, Donner A. Extension of the modified Poisson regression model to prospective studies with correlated binary data. Stat Methods Med Res. 2013 Dec 1;22(6):661–70.

14. Bai L, Gillen DL, Bartell SM, Vieira VM. Mapping Smoothed Spatial Effect Estimates from Individual-Level Data: MapGAM. | EBSCOhost [Internet]. Vol. 12. 2020 [cited 2024 Dec 9]. p. 32. Available from: https://openurl.ebsco.com/contentitem/doi:10.32614%2Frj-2020-001?sid=ebsco:plink:crawler&id=ebsco:doi:10.32614%2Frj-2020-001

15. Bai L, Gillen DL, Bartell SM, Vieira VM. Mapping Smoothed Spatial Effect Estimates from Individual-Level Data: MapGAM. R J. 2020;12(1):32–48.

16. Webster T, Vieira V, Weinberg J, Aschengrau A. Method for mapping population-based case-control studies: an application using generalized additive models. Int J Health Geogr. 2006 Jun 9;5:26.

17. Ugwu CLJ, Ncayiyana JR. Spatial disparities of HIV prevalence in South Africa. Do sociodemographic, behavioral, and biological factors explain this spatial variability? Front Public Health. 2022 Nov 11;10:994277.

18. Yilema SA, Shiferaw YA, Belay AT, Belay DB. Mapping the spatial disparities of HIV prevalence in Ethiopian zones using the generalized additive model. Sci Rep. 2024 Mar 14;14(1):6215.

19. Vieira V, Webster T, Weinberg J, Aschengrau A, Ozonoff D. Spatial analysis of lung, colorectal, and breast cancer on Cape Cod: An application of generalized additive models to case-control data. Environ Health. 2005 Jun 14;4(1):11.

20. Croft TN, Allen CK, Zachary BW. Guide to DHS Statistics [Internet]. Rockville Maryland, USA: ICF; 2023. Available from: https://dhsprogram.com

21. Lumley T. Analysis of Complex Survey Samples. J Stat Softw. 2004 Apr 15;9:1–19.
