# Supplementary material for: Trends and geographic patterns of overweight and obesity among Tanzanian adults: Evidence from the 2010–2022 Demographic and Health Surveys
Source: PLoS One. 2025 Sep 22;20(9):e0332275. doi: 10.1371/journal.pone.0332275 (PMC12453257; doi:10.1371/journal.pone.0332275)
Supplement: S2 Table — (DOCX) [file pone.0332275.s002.docx]

**S2 Table**: Distribution of participants across socio-demographic factors

| **Factor** | **2010** | | | **2015** | | **2022** | | | | |
| --- | --- | --- | --- | --- | --- | --- | --- | --- | --- | --- |
|  | **Women** | | | **Women** | | **Women** | | **Men** | |  |
|  | **Percentage (%)** | **n(weighted)** | **Percentage (%)** | | **n(weighted)** | **Percentage (%)** | **n(weighted)** | **Percentage (%)** | **n(weighted)** |  |
| **Age group** |  |  |  | |  |  |  |  |  |  |
| 15 - 19 | 21.90 | 1992 | 22.04 | | 2632 | 20.98 | 1480 | 25.06 | 1444 |  |
| 20 - 29 | 33.46 | 3042 | 33.33 | | 3981 | 33.28 | 2348 | 30.96 | 1784 |  |
| 30 - 39 | 26.62 | 2421 | 25.49 | | 3045 | 25.57 | 1804 | 25.31 | 1458 |  |
| 40 - 49 | 18.01 | 1638 | 19.14 | | 2286 | 20.18 | 1423 | 18.67 | 1076 |  |
|  |  |  |  | |  |  |  |  |  |  |
| **Residence** |  |  |  | |  |  |  |  |  |  |
| Rural | 70.38 | 6400 | 63.19 | | 7548 | 64.15 | 4525 | 66.37 | 3825 |  |
| Urban | 29.62 | 2693 | 36.81 | | 4397 | 35.85 | 2529 | 33.63 | 1938 |  |
|  |  |  |  | |  |  |  |  |  |  |
| **Marital Status** |  |  |  | |  |  |  |  |  |  |
| Never | 26.99 | 2454 | 26.90 | | 3214 | 28.98 | 2044 | 43.68 | 2517 |  |
| In union | 60.79 | 5527 | 59.66 | | 7127 | 57.93 | 4087 | 50.97 | 2937 |  |
| Separated | 12.22 | 1111 | 13.43 | | 1604 | 13.09 | 924 | 5.36 | 309 |  |
|  |  |  |  | |  |  |  |  |  |  |
| **Education** |  |  |  | |  |  |  |  |  |  |
| None | 18.37 | 1670 | 14.31 | | 1709 | 15.69 | 1107 | 9.96 | 574 |  |
| Primary | 64.26 | 5843 | 61.66 | | 7365 | 53.50 | 3774 | 54.38 | 3134 |  |
| Secondary | 16.91 | 1537 | 22.66 | | 2706 | 29.62 | 2090 | 32.24 | 1858 |  |
| Higher | 0.46 | 42 | 1.38 | | 165 | 1.19 | 84 | 3.42 | 197 |  |
|  |  |  |  | |  |  |  |  |  |  |
| **Occupation** |  |  |  | |  |  |  |  |  |  |
| None | 20.62 | 1875 | 23.18 | | 2768 | 36.24 | 2557 | 15.11 | 871 |  |
| Not manual | 2.47 | 225 | 3.82 | | 456 | 14.42 | 1017 | 13.15 | 758 |  |
| Manual | 76.91 | 6994 | 73.01 | | 8721 | 44.69 | 3153 | 66.53 | 3834 |  |
| Other | - | - | - | | - | 4.64 | 328 | 5.20 | 300 |  |
|  |  |  |  | |  |  |  |  |  |  |
| **Wealth index** |  |  |  | |  |  |  |  |  |  |
| Poorest | 16.38 | 1490 | 16.24 | | 1939 | 14.70 | 1037 | 15.32 | 883 |  |
| Poorer | 18.51 | 1683 | 16.79 | | 2005 | 17.30 | 1220 | 17.99 | 1037 |  |
| Middle | 19.14 | 1740 | 17.64 | | 2107 | 19.92 | 1405 | 20.67 | 1191 |  |
| Richer | 21.21 | 1928 | 21.50 | | 2568 | 22.38 | 1579 | 23.51 | 1355 |  |
| Richest | 24.76 | 2252 | 27.84 | | 3325 | 25.70 | 1813 | 22.52 | 1298 |  |
|  |  |  |  | |  |  |  |  |  |  |
| **Contraceptives** |  |  |  | |  |  |  |  |  |  |
| No | 67.92 | 6176 | 64.20 | | 7669 | 66.30 | 4677 |  |  |  |
| Yes | 32.08 | 2917 | 35.80 | | 4276 | 33.70 | 2377 |  |  |  |
|  |  |  |  | |  |  |  |  |  |  |
