# Supplementary material for: Trends and geographic patterns of overweight and obesity among Tanzanian adults: Evidence from the 2010–2022 Demographic and Health Surveys
Source: PLoS One. 2025 Sep 22;20(9):e0332275. doi: 10.1371/journal.pone.0332275 (PMC12453257; doi:10.1371/journal.pone.0332275)
Supplement: S3 Table — (DOCX) [file pone.0332275.s003.docx]

**S3 Table**: Comparisons of unadjusted and adjusted variable effects on overweight and obesity.

|  | **Model** | **Optimal span size** | **Residual**  **Deviance** | **AIC** | **p-value** | **Variable effect predictions (Prevalence ratio)** | | |
| --- | --- | --- | --- | --- | --- | --- | --- | --- |
|  |  |  |  |  |  | **Minimum** | **Mean** | **Maximum** |
| **Women**  **2010** | Unadjusted | 0.05 | 5372.02 | 9460.53 | <0.001 | 0.17 | 1.07 | 4.27 |
|  | Adjusted for all covariates | 0.1 | 4857.51 | 8895.29 | <0.001 | 0.44 | 0.99 | 1.88 |
|  | **Adjusted individual variables** |  |  |  |  |  |  |  |
|  | Age group | 0.05 | 5107.84 | 9202.51 | <0.001 | 0.17 | 1.08 | 4.20 |
|  | Residence | 0.1 | 5345.53 | 9353.32 | <0.001 | 0.44 | 1.02 | 2.75 |
|  | Marital Status | 0.05 | 5206.32 | 9298.97 | <0.001 | 0.17 | 1.07 | 4.50 |
|  | Education | 0.05 | 5359.05 | 9453.52 | <0.001 | 0.17 | 1.07 | 4.07 |
|  | Occupation | 0.05 | 5273.46 | 9366.00 | <0.001 | 0.17 | 1.07 | 4.07 |
|  | Wealth index | 0.1 | 5196.87 | 9210.59 | <0.001 | 0.45 | 1.01 | 1.85 |
|  | Contraceptives | 0.05 | 5315.50 | 9406.02 | <0.001 | 0.18 | 1.05 | 3.78 |
|  |  |  |  |  |  |  |  |  |
| **Women**  **2015** | Unadjusted | 0.05 | 8005.54 | 14902.15 | <0.001 | 0.29 | 1.02 | 4.47 |
|  | Adjusted for all covariates | 0.3 | 7014.45 | 13806.53 | <0.001 | 0.61 | 0.99 | 1.35 |
|  | **Adjusted individual variables** |  |  |  |  |  |  |  |
|  | Age group | 0.05 | 7381.59 | 14283.97 | <0.001 | 0.29 | 1.02 | 4.54 |
|  | Residence | 0.1 | 7950.04 | 14764.63 | <0.001 | 0.50 | 1.01 | 1.82 |
|  | Marital Status | 0.05 | 7680.10 | 14580.47 | <0.001 | 0.26 | 1.03 | 5.05 |
|  | Education | 0.05 | 7989.11 | 14891.81 | <0.001 | 0.31 | 1.02 | 4.38 |
|  | Occupation | 0.05 | 7861.25 | 14761.79 | <0.001 | 0.30 | 1.03 | 4.36 |
|  | Wealth index | 0.3 | 7749.30 | 14517.43 | <0.001 | 0.58 | 0.99 | 1.33 |
|  | Contraceptives | 0.05 | 7889.43 | 14788.12 | <0.001 | 0.30 | 1.03 | 4.28 |
|  |  |  |  |  |  |  |  |  |
| **Women**  **2022** | Unadjusted | 0.1 | 4965.64 | 9673.86 | <0.001 | 0.51 | 1.01 | 1.80 |
|  | Adjusted for all covariates | 0.95 | 4366.80 | 9039.96 | <0.001 | 0.78 | 0.99 | 1.14 |
|  | **Adjusted individual variables** |  |  |  |  |  |  |  |
|  | Age group | 0.2 | 4596.41 | 9273.29 | <0.001 | 0.70 | 1.03 | 1.62 |
|  | Residence | 0.2 | 4902.44 | 9575.32 | <0.001 | 0.68 | 1.01 | 1.50 |
|  | Marital Status | 0.1 | 4794.07 | 9506.31 | <0.001 | 0.50 | 1.01 | 1.87 |
|  | Education | 0.1 | 4950.49 | 9664.70 | <0.001 | 0.51 | 1.02 | 1.74 |
|  | Occupation | 0.2 | 4905.35 | 9582.19 | <0.001 | 0.73 | 1.03 | 1.63 |
|  | Wealth index | 0.35 | 4808.7 0 | 9469.87 | <0.001 | 0.78 | 1.01 | 1.25 |
|  | Contraceptives | 0.2 | 4957.81 | 9630.67 | <0.001 | 0.72 | 1.03 | 1.66 |
|  |  |  |  |  |  |  |  |  |
| **2022**  **Men** | Unadjusted | 0.25 | 3197.14 | 4975.40 | <0.001 | 0.55 | 0.99 | 1.65 |
|  | Adjusted for all covariates | 0.95 | 2757.78 | 4542.99 | 0.003 | 0.68 | 0.97 | 1.13 |
|  | **Adjusted individual variables** |  |  |  |  |  |  |  |
|  | Age group | 0.25 | 2939.88 | 4724.13 | <0.001 | 0.62 | 1.01 | 1.68 |
|  | Residence | 0.25 | 3157.47 | 4937.71 | <0.001 | 0.57 | 0.98 | 1.49 |
|  | Marital Status | 0.25 | 2989.29 | 4771.57 | <0.001 | 0.59 | 1.01 | 1.80 |
|  | Education | 0.3 | 3170.95 | 4948.66 | <0.001 | 0.60 | 0.97 | 1.53 |
|  | Occupation | 0.25 | 3072.82 | 4857.06 | <0.001 | 0.57 | 0.99 | 1.66 |
|  | Wealth index | 0.9 | 3051.82 | 4814.07 | <0.001 | 0.65 | 0.98 | 1.21 |

The spatial effect test indicates that in the unadjusted models, overweight and obesity was significantly associated with participants’ spatial locations (latitude and longitude) across all surveys (p-value < 0.05) (S3 Table). The optimal span size which minimized the AIC, increased over time from 0.05 in 2010 to 0.1 in 2022 for women, meaning that the proportion of adjacent neighbourhood data used for spatial smoothing was 5% and 10%, respectively. Adjusting for all risk factors led to further increase in the span size from 5% to 10% in 2010 and 2015, and from 10% to 95% in 2022 for women, as well as from 25% to 95% for men in 2022. These adjustments minimized AIC values, reduced residual deviance, and influenced predictions for all variables’ effects. The p-value for the global spatial effect remained statistically significant (p < 0.05). The unadjusted models revealed substantial geographic disparities in prevalence ratios, particularly in 2010 which ranged between 0.17 and 4.27 and in 2015 which ranged between 0.29 and 4.47. Over time, these disparities diminished (range narrowed to 0.51-1.80 in 2022 for women), indicating reduced spatial variability. Adjusting for all covariates, the prevalence ratio range became significantly narrower in each survey, with values ranging from 0.44-1.88 in 2010, 0.61-1.35 in 2015, 0.78-1.14 for women in 2022, and 0.68-1.13 for men in 2022 (Table 3). This reduced disparity was clearly depicted in Fig 4. Adjusting for individual covariates revealed their distinct contributions to overweight and obesity (S3 Table). Residence and wealth index consistently reduced the prevalence ratio range compared to other covariates. Age produced the smallest AIC values compared to unadjusted models and was associated with a wider prevalence ratio range in all surveys.
